# Forecasting the daily and cumulative number of cases for the COVID-19 pandemic in India

**DOI:** 10.1063/5.0016240

**Published:** 2020-07-08

**Authors:** Subhas Khajanchi, Kankan Sarkar

**Affiliations:** 1 Department of Mathematics, Presidency University, 86/1 College Street, Kolkata 700073, India; 2 Department of Mathematics, Malda College, Malda, West Bengal 732101, India

## Abstract

The ongoing novel coronavirus epidemic was announced a pandemic by the World Health Organization on March 11, 2020, and the Government of India declared a nationwide lockdown on March 25, 2020 to prevent community transmission of the coronavirus disease (COVID)-19. Due to the absence of specific antivirals or vaccine, mathematical modeling plays an important role in better understanding the disease dynamics and in designing strategies to control the rapidly spreading infectious disease. In our study, we developed a new compartmental model that explains the transmission dynamics of COVID-19. We calibrated our proposed model with daily COVID-19 data for four Indian states, namely, Jharkhand, Gujarat, Andhra Pradesh, and Chandigarh. We study the qualitative properties of the model, including feasible equilibria and their stability with respect to the basic reproduction number $R_{0}$. The disease-free equilibrium becomes stable and the endemic equilibrium becomes unstable when the recovery rate of infected individuals increases, but if the disease transmission rate remains higher, then the endemic equilibrium always remains stable. For the estimated model parameters, $R_{0}>1$ for all four states, which suggests the significant outbreak of COVID-19. Short-time prediction shows the increasing trend of daily and cumulative cases of COVID-19 for the four states of India.

In India, 173 763 confirmed cases, 7964 confirmed new cases, and 4971 confirmed deaths due to coronavirus disease (COVID)-19 were reported as of May 30, 2020. As the ongoing COVID-19 outbreak is quickly spreading throughout India and the world, short-term modeling predictions give time-critical statistics for decisions on containment and mitigation policies. A big problem in the short-term prediction is the evaluation of important parameters and how they alter when the first interventions reveal an effect. In the absence of any therapeutics or licensed vaccine and antivirals, isolation of population diagnosed with COVID-19 and quarantine of population feared exposed to COVID-19 were used to control the rapid spread of the infection. During this alarming situation, forecasting is of utmost priority for healthcare planning and to control the severe acute respiratory syndrome coronavirus (SARS-CoV)-2 virus with limited resources. We proposed a mathematical model that monitors the dynamics of six compartments, namely, susceptible (*S*), asymptomatic (*A*), reported symptomatic (*I*), unreported symptomatic (*U*), quarantine (*Q*), and recovered (*R*) individuals, collectively termed SAIUQR, which predicts the course of the epidemic. Our SAIUQR model discriminates between reported and unreported infected individuals, which is important as the former are typically isolated and, hence, less likely to spread the infection. A detailed theoretical analysis has been done for our SAIUQR model in terms of the basic reproduction number $R_{0}$. All analytical findings are verified numerically for the estimated model parameters. We have calibrated our SAIUQR model with real observed data on the COVID-19 outbreak in four states of India. The basic reproduction number for all states is greater than unity, which resulted in a substantial outbreak of COVID-19. Based on the simulation, our SAIUQR model predicts that on June 13, 2020, the daily new COVID-19 cases will be around 15, 454, 12, and 96, and the cumulative number of COVID-19 cases will be around 661, 23 955, 514, and 4487 in Jharkhand, Gujarat, Chandigarh, and Andhra Pradesh, respectively.

## I. INTRODUCTION

After a novel strain of COVID-19 was detected in Wuhan, the city of Hubei province, China, in December 2019,^1^ an exponentially increasing number of patients in mainland China were identified with SARS-CoV-2; immediately, the Chinese Health authorities initiated radical measures to control the epidemic coronavirus. In spite of these radical measures, the SARS-CoV-2 coronavirus pandemic ensued in the subsequent months and China became the epicenter. SARS-CoV-2 viruses are enveloped non-segmented positive-sense RNA viruses that belong to the Coronaviridae family and the order Nidovirales and are extensively disseminated among humans as well as mammals.^2^ COVID-19 is responsible for a range of symptoms together with fever, dry cough, breathing difficulties, fatigue, and lung infiltration in severe cases, similar to those created by SARS-CoV (severe acute respiratory syndrome coronavirus) and MERS-CoV (Middle East respiratory syndrome coronavirus) infections.^3^ SARS-CoV-2 has already crossed the earlier history of two coronavirus epidemics, SARS-CoV and MERS-CoV, posing substantial threat to the world population with health problems as well as economic problems after the Second World War.^4^ According to the World Health Organization report dated May 29, 2020, 5 596 550 total cases and 353 373 deaths were reported worldwide.^5^

To date, there are no licensed vaccines, drugs, and effective therapeutics available for SARS-CoV-2 or COVID-19. Due to the absence of pharmaceutical interventions, governments of various countries are adopting different strategies to control the outbreak, and the most common one is the nationwide lockdown. It was started by the local Government of Wuhan by temporarily locking down the city to prevent all public traffics within the city on January 23, 2020 and soon followed by other cities in Hubei province.^6^ In the absence of drugs or specific antivirals for SARS-CoV-2 virus, maintaining social distancing is the only way to mitigate the human-to-human transmission of the coronavirus disease, and thus other countries also incorporated strict lockdowns, quarantines, and curfews.

In India, the first coronavirus case was reported in Kerala’s Thrissur district on January 30, 2020, when a student returned from Wuhan, the sprawling capital of China’s Hubei province.^7^ The Government of India has implemented a complete nationwide lockdown throughout the country on and from March 25, 2020 for 21 days and one day “Janata Curfew” on March 22, 2020 to control the coronavirus or SARS-CoV-2 pandemic in India.^8^ Due to the massive spread of the coronavirus disease, the Government of India has extended the lockdown and it is in Phase 4 (from May 18, 2020 to May 31, 2020). Besides the implementation of nationwide lockdown, the Ministry of Health and Family Welfare (MOHFW) of India recommended different individual hygiene measures, for example, frequent hand washing, social distancing, use of mask, avoiding gatherings, touching of eyes, mouth, and nose, etc.^9^

The government also ceaselessly used different media and social networks to create awareness among the public regarding the coronavirus disease and its precautions. However, the factors such as diverse and huge population, the unavailability of specific therapeutics, drugs, or licensed vaccines, and inadequate evidence regarding the mechanism of disease transmission make it strenuous to combat against the coronavirus disease throughout India. To control the transmission of COVID-19, lockdown is a great measure, but testing is also an important factor to identify the symptomatic and asymptomatic individuals. The symptomatic individuals should be reported to the public health agencies to separate them from the uninfected or asymptomatic individuals for their ICU (Intensive Care Unit) treatment. Also, from an economic viewpoint, a strict lockdown could lead to substantial financial crisis in the near future. In particular, the lockdown in high density countries can mitigate the disease transmission rate, although it may not entirely control the disease. Thus, for a country to survive the economic status, a strict lockdown for a longer period is not advisable. Hence, there should be an acceptable balance between two different characteristics of governmental strategies: strict lockdown and healthy economic situation. However, few questions remain unanswered: whether this cluster containment policy can be effective in mitigating the SARS-CoV-2 transmission or not? If not, then what can be the possible solution to mitigate the transmission of the SARS-CoV-2 virus? These questions can only be answered by investigating the dynamics and forecasting of the SARS-CoV-2 transmission by a mechanistic compartmental model and comparing the outcomes with real scenarios.

Numerous mathematical models have been investigated to study the transmission dynamics and forecasting of the COVID-19 outbreak.^10–20^ Kucharski *et al.*^10^ performed a model-based analysis for SARS-CoV-2 virus and calculated the reproduction number $R_{0}=2.35$, where the authors have taken into account all positive cases of Wuhan, China, until March 5, 2020. Wu *et al.*^12^ studied a susceptible–exposed–infectious–recovered (SEIR) model to simulate the epidemic in Wuhan city and computed the basic reproduction number $R_{0}=2.68$; they predicted their model based on the data recorded from December 31, 2019 to January 28, 2020. Tang *et al.*^11^ developed a compartmental model to study the transmission dynamics of COVID-19 and calculated the basic reproduction number $R_{0}=6.47$, which is very high for an infectious disease. Recently, Fanelli and Piazza^13^ analyzed and predicted the characteristics of SARS-CoV-2 virus in three most affected countries until March 2020 by using the mathematical modeling. Ribeiro *et al.*^14^ used a stochastic based regression model to predict the scenarios of the most affected states of Brazil. Chakraborty and Ghosh^15^ investigated a hybrid ARIMA-WBF model to predict various SARS-CoV-2 affected countries throughout the world. Khajanchi *et al.*^18^ developed a compartmental model to forecast and control the outbreak of COVID-19 in four states of India and overall India. Sarkar and Khajanchi^16^ developed a mathematical model to study the model dynamics and forecast the SARS-CoV-2 virus in 17 states of India and overall India. A discrete-time SIR model introducing a dead compartment system was studied by Anastassopoulou *et al.*^19^ to portray the dynamics of the SARS-CoV-2 outbreak. Giordano *et al.*^20^ established a new mathematical model for the COVID-19 pandemic and predicted that restrictive social distancing can mitigate the widespread of COVID-19 among the humans. A couple of seminal papers were investigated to study the transmission dynamics of COVID-19 or SARS-CoV-2 virus in different countries, including Mexico city, Chicago, and Wuhan, the sprawling capital of Central China’s Hubei province.^21–24^ Short-term prediction is too important as it gives time-critical information for decisions on containment and mitigation strategies.^18,25^ A major problem for short-term predictions is the evaluation of important epidemiological parameters, and how they alter when first intervention reveals an effect.

The main objective of this work is to develop a new mathematical model that describes the transmission dynamics and forecasting of COVID-19 or SARS-CoV-2 pandemic in four different states of India, namely, Jharkhand, Andhra Pradesh, Chandigarh, and Gujarat. We estimated the model parameters of the four different states of India and fitted our compartmental model to the daily confirmed cases and cumulative confirmed cases reported between March 15, 2020 and May 24, 2020. We computed the basic reproduction number $R_{0}$ for the states based on the estimated parameter values. We also performed short-term predictions for the four states of India from May 25, 2020 to June 13, 2020, and it shows the increasing trends of COVID-19 pandemic in the four states.

The remaining part of this article has been organized in the following way. In Sec. II, we describe the formulation of the compartmental model for COVID-19 and its basic assumptions. Section III describes the theoretical analysis of the model, which incorporates the positivity and boundedness of the system, computation of the basic reproduction number $R_{0}$, and the existence of the biologically feasible singular points and their local stability analysis. In the same section, we perform the global stability analysis for the infection-free equilibrium point $E^{0}$ and the existence of transcritical bifurcation at threshold $R_{0}=1.$ In Sec. IV, we conduct some model simulations to validate our analytical findings by using the estimated model parameters for Jharkhand, a state of India. The parameters are estimated for the real world example on COVID-19 for four different states of India, and a short-term prediction based on the estimated parameter values was performed. A discussion in Sec. V concludes the article.

## II. MATHEMATICAL MODEL

A compartmental mathematical model has been developed to study the transmission dynamics of COVID-19 outbreak in India and throughout the world. We adopt a variant that focuses some important epidemiological properties of the COVID-19 or SARS-CoV-2 coronavirus disease. Based on the health status, we stratify the total human population into six compartments, namely, susceptible or uninfected (S), asymptomatic or pauci-symptomatic infected (A), symptomatic reported infected (I), unreported infected (U), quarantine (Q), and recovered (R) individuals, collectively termed SAIUQR. At any instant of time, the total population is denoted by N=S+A+I+U+Q+R. Depending on the six state variables, we aim to develop an autonomous system using first order nonlinear ordinary differential equations.

In the model formulation, quarantine refers to the separation of coronavirus infected population from the general population when the individuals are infected but clinical symptoms has not yet been developed, whereas isolation refers to the separation of coronavirus infected population when the population already identified the clinical symptoms. Our mathematical model introduces some demographic effects by assuming a proportional natural mortality rate δ>0 in each of the six compartments. In addition, our model incorporates a constant recruitment of susceptible populations into the region at the rate $\Lambda_{s}$ per unit time. This parameter represents new birth, immigration, and emigration. The parameter $\beta_{s}$ represents the probability of the disease transmission rate. However, for the disease transmission from vulnerable to infected individuals (for our model), the class is (*A*) depending on various factors, namely, safeguard precautions (use of mask, social distancing, etc.) and hygienic safeguard (use of hand sanitizer) taken by the susceptible individuals as well as infected population. In our model formulation, we incorporate the asymptomatic or pauci-symptomatic infected (undetected) individuals, which is important to better understand the transmission dynamics of COVID-19, which was also studied by Giordano *et al.*^20^ and Xiao-Lin *et al.*^21^

In our model formulation, we assumed that the COVID-19 virus spreads when a vulnerable person comes into contact with an asymptomatic infected individual. The uninfected individuals decreases after infection, obtained through interplays between a susceptible population and an infected individuals who may be asymptomatic, reported symptomatic, and unreported symptomatic. For these three compartments of infected population, the transmission coefficients are $\beta_{s}\alpha_{a}$, $\beta_{s}\alpha_{i}$, and $\beta_{s}\alpha_{u}$, respectively. We consider $\beta_{s}$ as the disease transmission rate along with the adjustment factors for asymptomatic $\alpha_{a}$, reported symptomatic $\alpha_{i}$, and unreported symptomatic $\alpha_{u}$ individuals. The interplays among infected populations (asymptomatic, reported symptomatic, and unreported symptomatic) and susceptible individuals can be modeled in the form of total individuals using standard mixing incidence.^26–29^

The quarantined population can either move to the susceptible or infected compartment (reported and unreported), depending on whether they are infected or not,^30^ with a portion $\rho_{s}$. Here, $\gamma_{q}$ is the rate at which the quarantined uninfected contacts were released into the wider community. Asymptomatic individuals were exposed to the virus but clinical symptoms of SARS-CoV-2 virus has not yet been developed. The asymptomatic individuals decreases due to contact with reported and unreported symptomatic individuals at the rate $\gamma_{a}$ with a portion θ∈(0,1), and become quarantine at the rate $\xi_{a}$. Also, the asymptomatic individuals recover at the rate $\eta_{a}$ and have a natural mortality rate δ. A fraction of quarantine individuals become reported infected individuals at the rate $\gamma_{q}$ with a portion $\rho_{s}$ [where $\rho_{s}\in (0,1)]$.

As we know, whether an individual is infected by the coronavirus disease or not can be identified by the RT-PCR screening test and a person with negative results with the RT-PCR screening test may yet be coronavirus positive as it may take around 7–21 days for the coronavirus symptoms to appear.^31^ Thus, a fraction of coronavirus positive class can be considered as reported symptomatic individuals (θ) and unreported symptomatic individuals (1−θ). The reported symptomatic individuals are separated from the general population and moved to the isolated class or hospitalized class for clinical treatment.

Also, it can be noticed that once an individual recovered from the SARS-CoV-2 disease, he has very little chance to become infected again for the same disease.^31^ Therefore, we assume that none of the recovered individuals move to the susceptible or uninfected class again. In our mathematical model formulation, we assume that the reported infected individuals (I) are unable to spread or transmit the virus as they are kept completely isolated from the susceptible or uninfected individuals, as the reported infected individuals are moved to the hospital or Intensive Care Unit (ICU).^32^ For our modeling perspective, we are mainly interested in predictions over a relatively short time window within which the temporary immunity is likely still to be in place, and the possibility of reinfection would negligibly affect the total number of uninfected populations and so there would be no considerable difference in the evolution of the epidemic curves we consider. Social mixing patterns are introduced into our contagion parameters in an average fashion over the entire individuals, irrespective of age. Based on these biological assumptions, we develop the following mathematical model using a system of nonlinear ordinary differential equations to study the outbreak of COVID-19 or SARS-CoV-2 coronavirus disease:

$$\begin{array}{r} \left\{ \begin{array}{l} S^{\prime }(t)=\Lambda_{s}-\beta_{s}S(\alpha_{a}\frac{A}{N}+\alpha_{i}\frac{I}{N}+\alpha_{u}\frac{U}{N})+\rho_{s}\gamma_{q}Q-\delta S,\\ A^{\prime }(t)=\beta_{s}S(\alpha_{a}\frac{A}{N}+\alpha_{i}\frac{I}{N}+\alpha_{u}\frac{U}{N})-(\xi_{a}+\gamma_{a})A-\eta_{a}A-\delta A,\\ I^{\prime }(t)=\theta \gamma_{a}A+(1-\rho_{s})\gamma_{q}Q-\eta_{i}I-\delta I,\\ U^{\prime }(t)=(1-\theta )\gamma_{a}A-\eta_{u}U-\delta U,\\ Q^{\prime }(t)=\xi_{a}A-\gamma_{q}Q-\delta Q,\\ R^{\prime }(t)=\eta_{u}U+\eta_{i}I+\eta_{a}A-\delta R. \end{array}\right. \end{array}$$ (1)

The model is supplemented by the following initial values:

$$\begin{array}{rl} S(t_{0}) & =S_{0}\geq 0,\;A(t_{0})=A_{0}\geq 0,\;Q(t_{0})=Q_{0}\geq 0,\\ I(t_{0}) & =I_{0}\geq 0,\;U(t_{0})=U_{0}\geq 0,\;R(t_{0})=R_{0}\geq 0. \end{array}$$ (2)

**FIG. 1.**
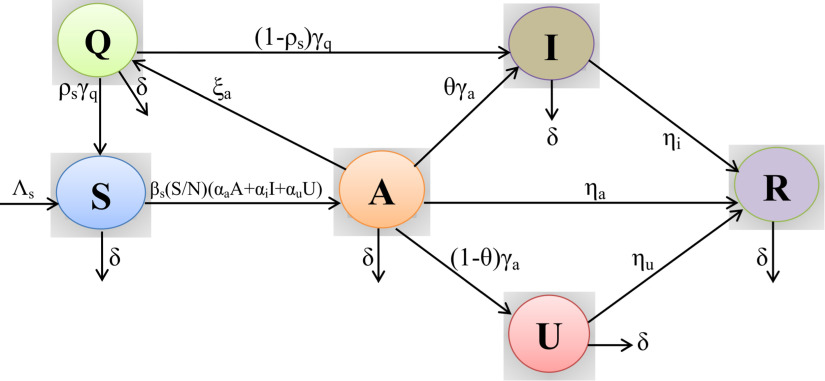
A schematic representation of the mechanistic SAIUQR model for the transmission dynamics of COVID-19 or SARS-CoV-2. The interaction among different stages of individuals is shown in the graphical scheme: S, susceptible or uninfected population; A, asymptomatic infected population; I, COVID-19 reported symptomatic infected individuals; U, COVID-19 unreported symptomatic infected individuals; Q, quarantine individuals; and R, COVID-19 recovered individuals. Biological interpretations of the model parameters are given in Table I.

In our model, $t\geq t_{0}$ is the time in days and $t_{0}$ represents the starting date of the outbreak for our system (1). The transmission dynamics of the COVID-19 is illustrated in Fig. 1. The description of the model parameters is presented in Table I.

**TABLE I.** Table of the biologically relevant parameter values and their description for the SAIUQR model system (1).

Symbol	Biological interpretations	Values and source
Λ_*s*_	Birth rate of the susceptible individuals	Table III
*β*_*s*_	Probability of the disease transmission coefficient	Estimated
*α*_*a*_	Modification factor for asymptomatic infected individuals	Estimated
*α*_*i*_	Modification factor for symptomatic infected individuals	Estimated
*α*_*u*_	Modification factor for unreported infected individuals	Estimated
*ρ*_*s*_	Fraction of quarantine individuals that become susceptible individuals	0.5 (0, 1), fixed
*γ*_*q*_	Rate at which the quarantined individuals becomes susceptible individuals	Estimated
*δ*	Natural death rate of all individuals	0.194 5 × 10^−4^^18^
*ξ*_*a*_	Rate at which the asymptomatic individuals become quarantined	0.071 51^16^
*γ*_*a*_	Rate of transition from the asymptomatic individuals to infected individuals	Estimated
*η*_*a*_	Average recovery rate of asymptomatic individuals	$\frac{1}{7.48}$^16^
*θ*	Fraction of asymptomatic individuals that become reported infected individuals	0.8 (0, 1), fixed
*η*_*i*_	Average recovery rate of reported symptomatic infected individuals	$\frac{1}{7}$^32^
*η*_*u*_	Average recovery rate of unreported symptomatic infected individuals	$\frac{1}{7}$^32^

## III. SAIUQR MODEL ANALYSIS

In this section, we provide the basic properties of the SAIUQR model (1), including positivity and boundedness of the solutions, basic reproduction number and the biologically feasible singular points and their stability analysis, subject to the non-negative initial values of $(S_{0},A_{0},Q_{0},I_{0},U_{0},Q_{0},R_{0})\in R^{6}.$

Theorem 3.1 The solutions of the SAIUQR system (1) with the initial values (2) are defined with $R_{+}^{6}$ remaining positive for all t>0.

Proof. The proof of this theorem is given in Appendix A.

Theorem 3.2 The solutions of the SAIUQR system (1) with the initial conditions of (2) are uniformly bounded in the region Ω.

Proof. The proof of this theorem is given in Appendix B.

### A. Basic reproduction number

In any infectious disease modeling, the basic reproduction number is the key epidemiological parameter for describing the characteristics of the disease. The basic reproduction number is symbolized by $R_{0}$ and is defined as “the number of secondary infected individuals caused by a single infected individual in the entire susceptible individuals.”^33^ The dimensionless basic reproduction number $R_{0}$ quantifies the expectation of the disease dying out or the spreading of the disease. $R_{0}<1$ describes, on an average, the infected population spread less than a new infection during the course of its infection period; thus, the disease can be cured. $R_{0}>1$ describes each infected individual spread on an average more than one new infection; thus, the disease can spread throughout the population. Various techniques can be used to compute the basic reproduction number $R_{0}$ for an epidemic outbreak. In this study, we use the next generation matrix to evaluate $R_{0}$.^33^ In our compartmental model, the following classes are explicitly related to the outbreak of the novel coronavirus disease: A, I, U, and Q. Thus, from the SAIUQR model system (1), we get the matrices F for the new infection and V for the transition terms, respectively, by

$$\begin{array}{rl} F & =\left[\begin{array}{c} \beta_{s}S(\alpha_{a}\frac{A}{N}+\alpha_{i}\frac{I}{N}+\alpha_{u}\frac{U}{N})\\ 0\\ 0\\ 0 \end{array}\right],\\ V & =\left[\begin{array}{c} (\xi_{a}+\gamma_{a}+\eta_{a}+\delta )A\\ -\theta \gamma_{a}A-(1-\rho_{s})\gamma_{q}Q+(\eta_{i}+\delta )I\\ -(1-\theta )\gamma_{a}A+(\eta_{u}+\delta )U\\ -\xi_{a}A+(\gamma_{q}+\delta )Q \end{array}\right]. \end{array}$$

The variational matrix for the SAIUQR system (1) can be evaluated at an infection-free singular point $E^{0}(S^{0},A^{0},Q^{0},I^{0},U^{0},R^{0})=(\frac{\Lambda_{s}}{\delta },0,0,0,0,0)$ by

$$\begin{array}{rl} F & =\left[\begin{array}{cccc} \beta_{s}\alpha_{a}\frac{S}{N} & \beta_{s}\alpha_{i}\frac{S}{N} & \beta_{s}\alpha_{u}\frac{S}{N} & 0\\ 0 & 0 & 0 & 0\\ 0 & 0 & 0 & 0\\ 0 & 0 & 0 & 0 \end{array}\right],\\ V & =\left[\begin{array}{cccc} \xi_{a}+\gamma_{a}+\eta_{a}+\delta & 0 & 0 & 0\\ -\theta \gamma_{a} & \left(\eta_{i}+\delta \right) & 0 & -(1-\rho_{s})\gamma_{q}\\ -(1-\theta )\gamma_{a} & 0 & \left(\eta_{u}+\delta \right) & 0\\ -\xi_{a} & 0 & 0 & \gamma_{q}+\delta \end{array}\right]. \end{array}$$

The basic reproduction number $R_{0}=\rho (FV^{-1})$, where $\rho (FV^{-1})$ represents the spectral radius for a next generation matrix $FV^{-1}$. Therefore, from the SAIUQR model (1), we get the basic reproduction number $R_{0}$ as

$$\begin{array}{rl} \rho (FV^{-1}) & =R_{0}\;=\;\frac{\beta_{s}\alpha_{a}}{\xi_{a}+\gamma_{a}+\eta_{a}+\delta }\\ & \;+\;\;\frac{(1-\theta )\beta_{s}\alpha_{u}\gamma_{a}}{\left(\eta_{u}+\delta )(\xi_{a}+\gamma_{a}+\eta_{u}+\delta \right)}\\ & \;+\frac{\beta_{s}\alpha_{i}(\theta \gamma_{a}(\gamma_{q}+\delta )+(1-\rho_{s})\xi_{a}\gamma_{q})}{\left(\xi_{a}+\gamma_{a}+\eta_{a}+\delta )(\gamma_{q}+\delta )(\eta_{i}+\delta \right)}. \end{array}$$

### B. Equilibria

The SAIUQR model (1) has two biologically feasible equilibrium points, namely,

(i) infection-free steady state $E^{0}(S^{0},A^{0},Q^{0},I^{0},U^{0},R^{0})=(\frac{\Lambda_{s}}{\delta },0,0,0,0,0)$, and
(ii) the endemic equilibrium point $E^{*}(S^{*},A^{*},Q^{*},I^{*},U^{*},R^{*})$, where $S^{*}=\frac{\Lambda_{s}}{\delta }-\hat{S}A^{*},\;I^{*}=\hat{I}A^{*},\;U^{*}\;=\;\hat{U}A^{*},\;Q^{*}\;=\;\hat{Q}A^{*}$, and $R^{*}\;=\;\hat{R}A^{*}$. The expression of $A^{*}$ is given by $A^{*}=\frac{\Lambda_{s}(R_{0}-1)}{\delta \left\{\hat{S}(R_{0}-1)+1+\hat{I}+\hat{U}+\hat{Q}+\hat{R}\right\}}$, where $\hat{S}\;=\;\frac{1}{\delta }\left[\xi_{a}\;+\;\gamma_{a}\;+\;\eta_{a}\;+\;\delta \;-\;\frac{\xi_{a}\rho_{s}\gamma_{q}}{\gamma_{q}+\delta }\right]$, $\hat{I\;}\;\;=\;\frac{\theta \gamma_{a}(\gamma_{q}+\delta )+(1-\rho_{s})\xi_{a}\gamma_{q}}{\left(\eta_{i}+\delta )(\gamma_{q}+\delta \right)}$, $\hat{U}\;=\;\frac{(1-\theta )\gamma_{a}}{\eta_{u}+\delta }$, $\hat{Q}\;=\;\frac{\xi_{a}}{\gamma_{q}+\delta }$, and $\hat{R}\;=\;\frac{1}{\delta }[\frac{(1-\theta )\gamma_{a}\eta_{u}}{\eta_{u}+\delta }\;+\;\frac{\eta_{i}\left\{\theta \gamma_{q}(\gamma_{q}\;+\;\delta )\;+\;(1-\rho_{s})\gamma_{q}\xi_{a}\right\}}{\left(\gamma_{q}+\delta )(\eta_{i}+\delta \right)}+\;\eta_{a}]$.

It can be observed the infection-free singular point $E^{0}(S^{0},A^{0},Q^{0},I^{0},U^{0},R^{0})$ is always feasible and the endemic equilibrium point $E^{*}(S^{*},A^{*},Q^{*},I^{*},U^{*},R^{*})$ is feasible if the following conditions hold:

$$\begin{array}{rl} \text{(i)}\; & R_{0}\;>\;1,\\ \text{(ii)}\; & \frac{1}{\xi_{a}+\gamma_{a}+\eta_{a}+\delta }\left[\frac{\Lambda_{s}}{A^{*}}+\frac{\xi_{a}\rho_{s}\gamma_{q}}{\gamma_{q}+\delta }\right]\;>\;1. \end{array}$$

### C. Stability analysis

In this subsection, we investigate the linear stability analysis for the SAIUQR model (1) for the two feasible steady states. By using the techniques of linearization, we investigate the local dynamics of the complicated system of the coronavirus compartmental model. Generally, we linearize the SAIUQR model around each of the feasible steady state and perturb the compartmental model by a very small amount and observe whether the compartmental model returns to that steady state or converges to any other steady state or attractor. The local stability analysis aids in understanding the qualitative behavior of the complex nonlinear dynamical system. By using the following theorem, we prove the local stability of the infection-free singular point $E^{0}(S^{0},A^{0},Q^{0},I^{0},U^{0},R^{0})$:

Theorem 3.3 The infection-free steady state $E^{0}$ is locally asymptotically stable if $R_{0}<1$ and unstable if $R_{0}>1$.

Proof. The proof of this theorem is given in Appendix C.

Theorem 3.4 The infection-free steady state $E^{0}$ is globally asymptotically stable for $R_{0}<1$ in the bounded region Ω.

Proof. The proof of this theorem is given in Appendix D.

Theorem 3.5 The SAIUQR model system (1) is locally asymptotically stable around the endemic equilibrium point $E^{*}$ for $R_{0}>1$. Also, system (1) experiences forward bifurcation at $R_{0}=1$.

Proof. The proof of this theorem is given in Appendix E.

## IV. NUMERICAL SIMULATION

In this section, we conduct some numerical illustrations to validate our analytical findings. Analytically, we perform the local stability analysis for infection-free steady state $E^{0}$ and a unique endemic equilibrium point $E^{*}$. We also perform the transcritical bifurcation at the threshold $R_{0}=1$ and the global stability analysis for disease-free steady state $E^{0}$. In order to validate the analytical calculations, we used the estimated parameter values for Jharkhand, the state of India and the techniques for parameter estimation are described in Subsection IV A.

### A. Model calibration

**FIG. 2.**
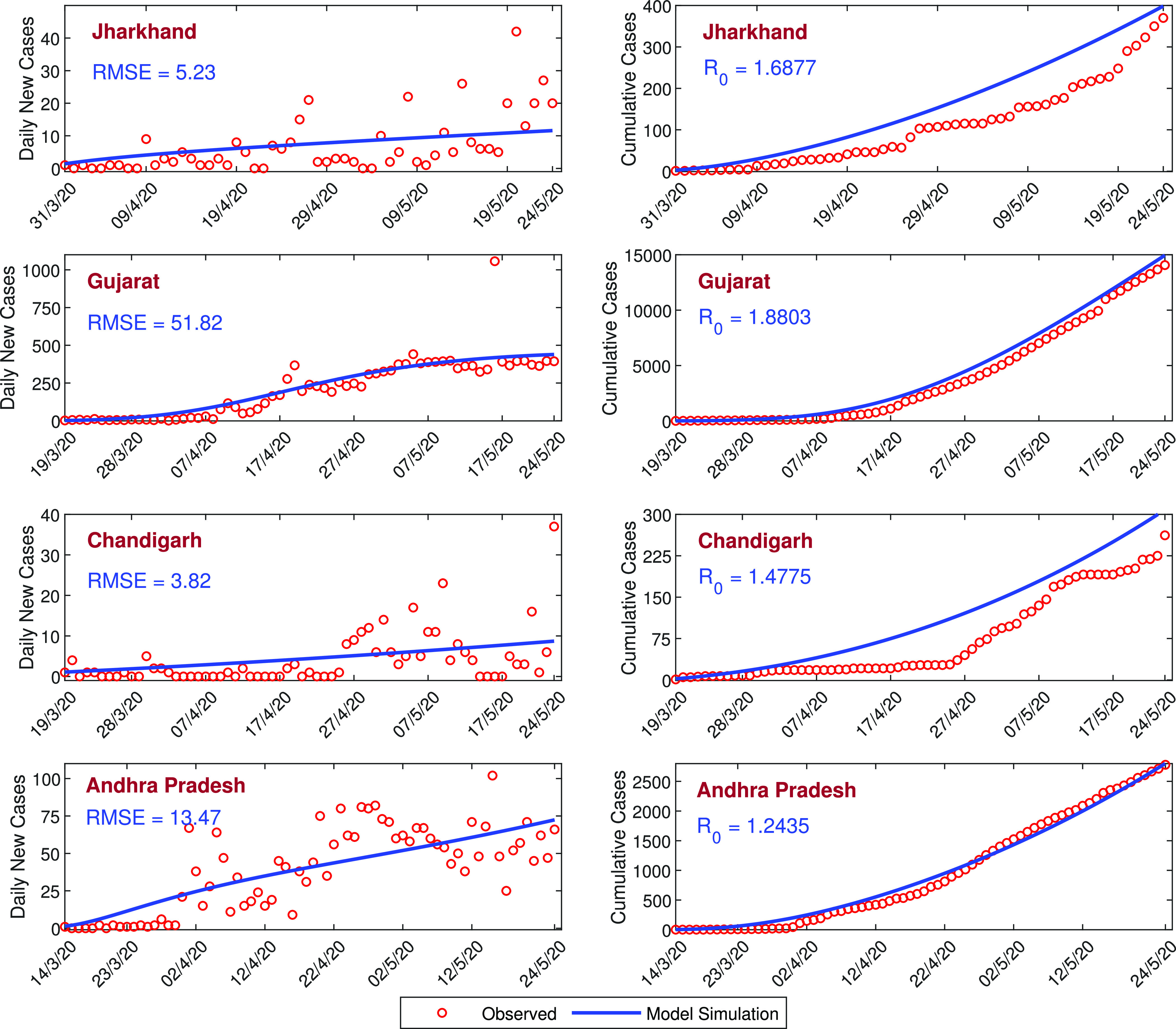
Model estimation based on the observed data. Model simulations fitted with the daily new cases and the cumulative confirmed cases of COVID-19 for four states of India, namely, Jharkhand, Gujarat, Chandigarh, and Andhra Pradesh. Observed data points are shown in the red circle, while the blue curve represents the best fitting curve for the SAIUQR model. The first column represents the daily new cases, and the second column represents the cumulative confirmed cases of COVID-19. The estimated parameter values are listed in Table II, and other parameter values are listed in Table I. The initial values used for this parameter values are presented in Table III. The RMSE and the value of $R_{0}$ for each states are mentioned in the inset.

We have calibrated our SAIUQR model (1) with the observed daily new COVID-19 cases. We have considered three states of India, namely, Jharkhand, Gujarat, Andhra Pradesh, and one city of India, namely, Chandigarh. The daily new COVID-19 cases are collected from the first COVID-19 case reported and up to May 24, 2020. The daily reported COVID-19 data were obtained from COVID19 INDIA (https://www.covid19india.org/).^34^ We have estimated six model parameters, namely, $\beta_{s}$, $\alpha_{a}$, $\alpha_{i}$, $\alpha_{u}$, $\gamma_{a}$, and $\gamma_{q}$, out of 14 system parameters for system (1) by using the least square method.^35^ The values of these parameters and the initial population size plays an important role in the model simulation. The parameters are estimated by assuming the initial population size. The initial population is presented in Table III. Three days moving average filter has been applied to the daily COVID-19 cases to smooth the data. The daily reported confirmed COVID-19 cases are fitted with the model simulation by using the least square method. The estimated parameter values are listed in Table II. Different sets of parameter values locally minimize the Root Mean Square Error (RMSE), and we have considered the set of parameter values, which gives the realistic value of the basic reproduction number $R_{0}$. RMSE is the measure of the accuracy of the fitting data and the RMSE is defined as follows:

$$\text{RMSE}=\sqrt{\frac{\Sigma_{i=1}^{n}{\left(O(i)-M(i)\right)}^{2}}{n}},$$

where n represents the size of the observed data, O(i) is the reported daily confirmed COVID-19 cases, and M(i) represents the model simulation. In Fig. 2, daily confirmed COVID-19 cases (first column), cumulative confirmed COVID-19 cases (second column), and model simulations have been shown by the blue curve for all four states of India. Values of RMSE and basic reproduction number $R_{0}$ for all four states are presented in the inset of the figure. The SAIUQR model performs well for the three states, namely, Jharkhand, Chandigarh, and Andhra Pradesh. The RMSE for Gujarat is higher than the other states as the number of daily confirmed COVID-19 cases are higher than the other states. The values of the basic reproduction number $R_{0}$ for Jharkhand, Gujarat, Chandigarh, and Andhra Pradesh are 1.6877, 1.8803, 1.4775, and 1.2435, respectively, and the trend of daily confirmed COVID-19 cases is increasing. This increasing trend of the daily new COVID-19 cases for all four states of India are captured by our model simulation. In all four states, $R_{0}>1$, so the disease-free equilibrium point $E^{0}$ is unstable. The basic reproduction numbers for the four states are greater than unity, which indicates the substantial outbreak of the COVID-19 in the states.

**TABLE II.** The SAIUQR model parameter values estimated from the observed daily new COVID-19 cases for four states of India, namely, Jharkhand, Gujarat, Chandigarh, and Andhra Pradesh. Six important parameters *β*_*s*_, *α*_*a*_, *α*_*i*_, *α*_*u*_, *γ*_*a*_, and *γ*_*q*_ are estimated among 14 system parameters.

Provinces	*β*_*s*_	*α*_*a*_	*α*_*i*_	*α*_*u*_	*γ*_*a*_	*γ*_*q*_
Jharkhand	0.760	0.264	0.760	0.9600	0.0012	0.0015
Gujarat	1.006	0.342	0.168	0.1308	0.0004	0.0046
Chandigarh	0.750	0.294	0.444	0.4600	0.0010	0.0011
Andhra Pradesh	0.431	0.419	0.688	0.7100	0.0006	0.0280

**TABLE III.** Initial population size and values of Λ_*s*_ used in numerical simulations for four different states of India, namely, Jharkhand, Gujarat, Chandigarh, and Andhra Pradesh.

Provinces	*S*(0)	*A*(0)	*Q*(0)	*I*(0)	*U*(0)	*R*(0)	Λ_*s*_
Jharkhand	39 402	575	19	1	0	0	1200
Gujarat	85 402	1525	27	1	0	0	1300
Chandigarh	20 402	275	10	1	0	0	1200
Andhra Pradesh	75 401	355	12	1	0	0	970

### B. Validation of analytical findings

**FIG. 3.**
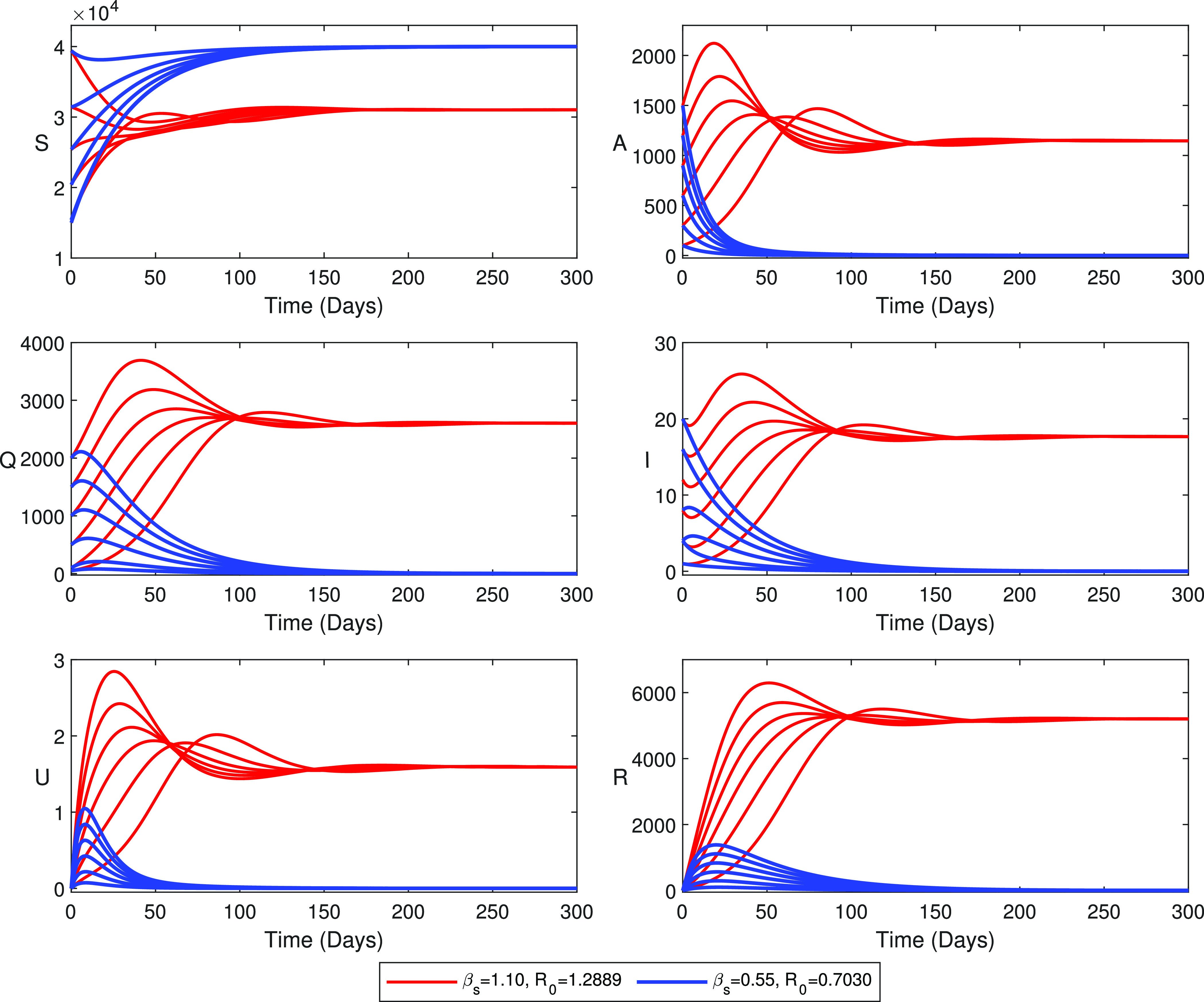
Stability of the SAIUQR model system (1) around the disease-free equilibrium point $E^{0}$ and an unique endemic equilibrium point $E^{*}$. Values of the estimated parameters are $\alpha_{a}=0.264,$ $\alpha_{i}=0.76,$ $\alpha_{u}=0.96,$ $\gamma_{a}=0.0012,$ $\gamma_{q}=0.0015,$ δ=0.03, and $\Lambda_{s}=1200$, and other parameter values are listed in Table I. Initial population sizes are (39402,1500,2000,20,0,0), (31402,1200,1500,16,0,0), (25402,900,1000,12,0,0), (20402,600,500,8,0,0), (15402,300,100,4,0,0), and (15000,100,50,1,0,0). Time series solution for $\beta_{s}=1.10$ (red curves) and $\beta_{s}=0.55$ (blue curves). Values of $R_{0}$ are 1.2889 and 0.7030 for $\beta_{s}=1.10$ and $\beta_{s}=0.55$, respectively. Disease-free equilibrium point $E^{0}$ is locally asymptotically stable when $R_{0}<1$ (blue curves), and the endemic equilibrium point $E^{*}$ is locally asymptotically stable when $R_{0}>1$ (red curves).

In this section, we have validated our analytical findings by using numerical simulations for the parameter values in Table I, and the estimated parameter values in Table II for our SAIUQR model for the coronavirus disease. The parameter values are estimated for the observed COVID-19 data for the three states of India, namely, Jharkhand, Gujarat, and Andhra Pradesh, and for the city Chandigarh. Our analytical findings stated in Theorem 3.3 show that the disease-free equilibrium point $E^{0}$ is locally asymptotically stable with $R_{0}<1$, and Theorem 3.5 stated that a unique endemic equilibrium point $E^{*}$ is locally asymptotically stable for $R_{0}>1$. The numerical simulations of the SAIUQR model system (1) have been presented in Fig. 3 for all six individuals and for the different values of the disease transmission rate $\beta_{s}$. The values of the parameters considered for numerical simulations are $\alpha_{a}=0.264,$ $\alpha_{i}=0.76,$ $\alpha_{u}=0.96,$ $\gamma_{a}=0.0012,$ $\gamma_{q}=0.0015,$ δ=0.03, $\Lambda_{s}=1200$, and the other model parameter values are listed in Table I. Six initial population sizes are considered for the model simulation, namely, (39402,1500,2000,20,0,0), (31402,1200,1500,16,0,0), (25402,900,1000,12,0,0), (20402,600,500,8,0,0), (15402,300,100,4,0,0), and (15000,100,50,1,0,0). The time series simulation has been displayed for $\beta_{s}=1.10$ (red curves in Fig. 3) and $\beta_{s}=0.55$ (blue curves in Fig. 3). Values of $R_{0}$ are 1.2889 and 0.7030 for $\beta_{s}=1.10$ and $\beta_{s}=0.55$, respectively. The blue curves in Fig. 3 show that the disease-free equilibrium point $E^{0}(40\;000,0,0,0,0,0)$ is locally asymptotically stable as well as globally asymptotically stable with $R_{0}=0.7030<1$. Our SAIUQR model system (1) converges to the endemic equilibrium point $E^{*}(31\;035.0,1146.0,17.6,1.6,2601.5,5198.4)$ for $\beta_{s}=1.10$ and $R_{0}=1.2889>1$ (red curves), which has been displayed in Fig. 3. Hence, this numerical simulation verifies the analytical findings in Theorems 3.3 and 3.5.

**FIG. 4.**
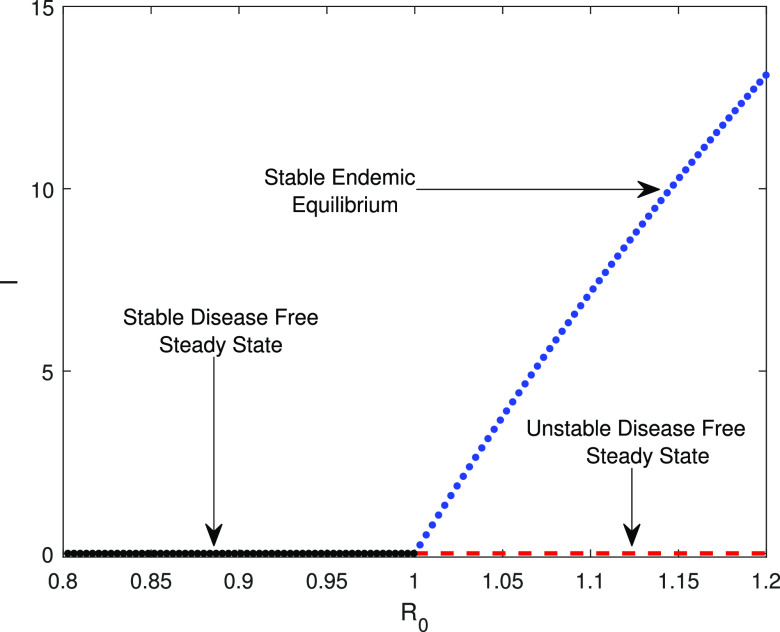
The transcritical bifurcation diagram of the SAIUQR model system (1) with respect to the basic reproduction number $R_{0}$. The parameter values are $\alpha_{a}=0.264,$ $\alpha_{i}=0.76,$ $\alpha_{u}=0.96,$ $\gamma_{a}=0.0012,$ $\gamma_{q}=0.0015,$ δ=0.03, and $\Lambda_{s}=1200$, and other parameters as listed in Table I. Stability of the SAIUQR system (1) exchange at the threshold $R_{0}=1$.

Theorem 3.5 states that the SAIUQR model system (1) undergoes a transcritical bifurcation at the threshold $R_{0}=1$. We have plotted the COVID-19 reported symptomatic individuals (I) in the $\left(R_{0},I\right)$ plane by gradually increasing the disease transmission rate $\beta_{s}$ (see Fig. 4). The model parameter values are $\alpha_{a}=0.264,$ $\alpha_{i}=0.76,$ $\alpha_{u}=0.96,$ $\gamma_{a}=0.0012,$ $\gamma_{q}=0.0015,$ δ=0.03, and $\Lambda_{s}=1200$, and other parameter values are listed in Table I. We vary the disease transmission rate $\beta_{s}$ from 0.67 to 1.10 and computed the basic reproduction number $R_{0}$ and the COVID-19 reported symptomatic individuals (I). The numerically computed values are presented in Fig. 4, which clearly shows that system (1) experiences transcritical bifurcation at the threshold $R_{0}=1$. The blue curve represents the stable endemic equilibrium point $E^{*}$, the black line represents the stable disease-free equilibrium point $E^{0}$, and the red line represents the unstable branch of the disease-free equilibrium point $E^{0}$. Hence, Fig. 4 shows that disease-free equilibrium point $E^{0}$ is stable for the reproduction number $R_{0}<1$ and an endemic equilibrium point $E^{*}$ is stable for the reproduction number $R_{0}>1$. From the biological point of view, it can be described that the model system (1) will be free from COVID-19 for the reproduction number $R_{0}<1$ and the coronavirus disease will spread throughout the people for $R_{0}>1.$

**FIG. 5.**
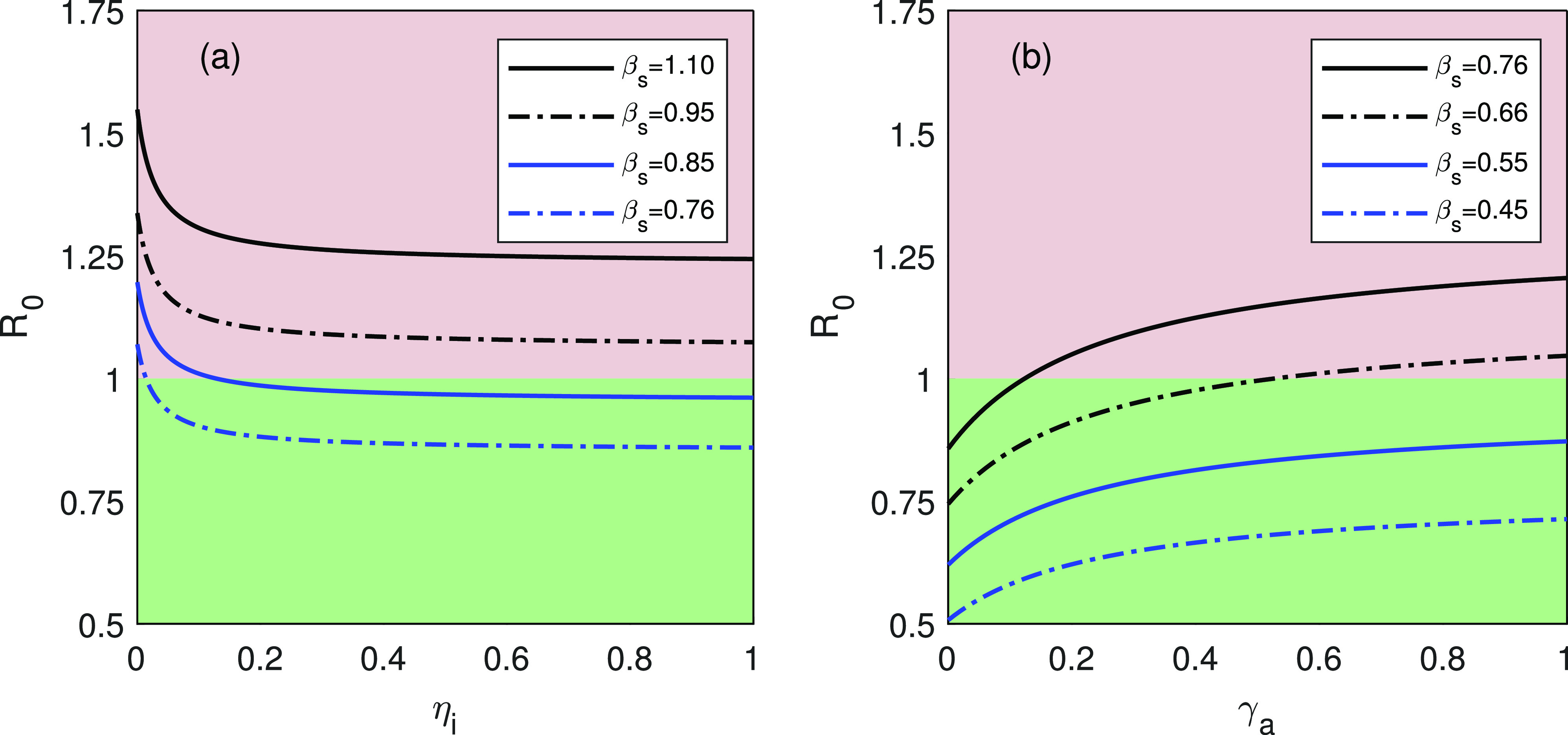
The basic reproduction number $R_{0}$ in terms of (a) $\eta_{i}$ (rate of recovery for infected individuals) and (b) $\gamma_{a}$ (rate at which asymptomatic individuals develops detected symptomatic infected individuals). The green shaded region indicates $R_{0}<1$, whereas the pink shaded region indicates $R_{0}>1$. The parameter values are $\alpha_{a}=0.264,$ $\alpha_{i}=0.76,$ $\alpha_{u}=0.96,$ $\gamma_{a}=0.0012,$ $\gamma_{q}=0.0015,$ δ=0.03, and $\Lambda_{s}=1200$, and other parameter values are listed in Table I.

Figure 5(a)  shows that the reproduction number $R_{0}$ decreases as the recovery rate $\eta_{i}$ of reported infected individuals increases and the reproduction number $R_{0}$ becomes less than one for $\beta_{s}=0.85$ and $\beta_{s}=0.76$. This indicates that the disease-free equilibrium point $E^{0}$ switches the stability of the model system (1) as $\eta_{i}$ changes. But the reproduction number $R_{0}$ remains greater than one for the disease transmission rates $\beta_{s}=1.10$ and $\beta_{s}=0.95$, that is, for large $\beta_{s}$, the unique endemic equilibrium point remains locally asymptotically stable even if $\eta_{i}$ changes. In terms of COVID-19 disease, this interprets that if the rate of recovery for infected individuals $\left(\eta_{i}\right)$ be increased, which can be done by vaccinees or specific therapeutics, the model system (1) changes its stability to disease-free equilibrium $E^{0}$ from endemic equilibrium $E^{*}$ but if the transmission of the disease $\left(\beta_{s}\right)$ is high enough, then by vaccines or specific therapeutics, system (1) cannot change its stability from endemic equilibrium to disease-free equilibrium.

Figure 5(b) shows that the reproduction number $R_{0}$ increases as $\gamma_{a}$ (transition rate from asymptomatic individuals to symptomatic individuals) increases but the reproduction number $R_{0}$ remains less than one for the disease transmission rates $\beta_{s}=0.55$ and $\beta_{s}=0.45$. For $\beta_{s}=0.66$ and $\beta_{s}=0.76$, the basic reproduction number $R_{0}$ becomes greater than one and the SAIUQR model system (1) loses the stability of disease-free equilibrium point $E^{0}$. Thus, to flatten the COVID-19 curve in any of the four states of India, reduction of the transmission of the COVID-19 disease is of utmost priority even if the recovery rate increased by medication. Biologically, it means that to mitigate the COVID-19 disease, the people must maintain social distancing and contact tracing by avoiding mass gatherings.

**FIG. 6.**
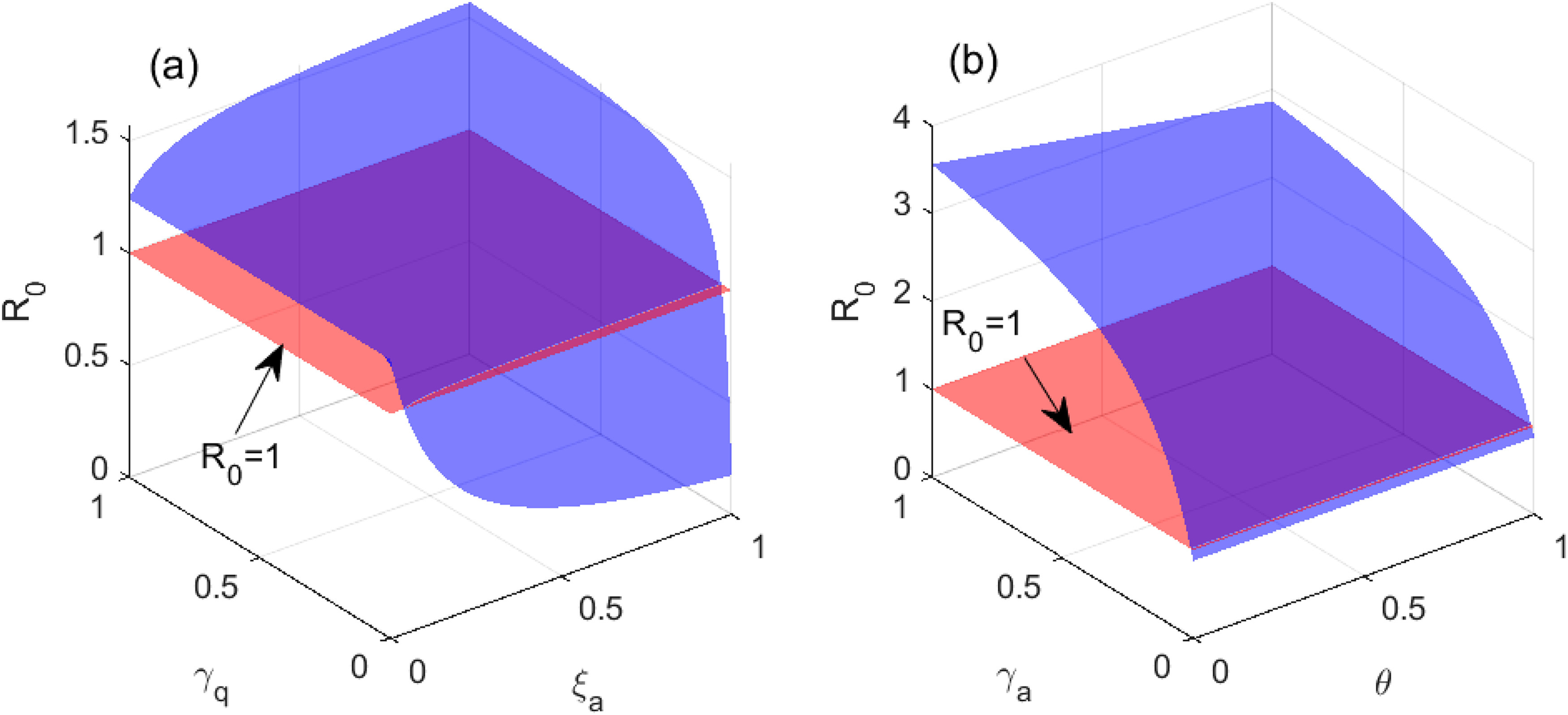
The surface plot of the basic reproduction number $R_{0}$ in (a) ($\gamma_{q}$,  $\xi_{a}$)-plane and (b) ($\gamma_{a}$,  θ)-plane. Red shading plane indicates $R_{0}=1$. The parameter values for sub-figure (a) are $\beta_{s}=0.76$, $\alpha_{a}=0.264,$ $\alpha_{i}=0.76,$ $\alpha_{u}=0.96,$ $\gamma_{a}=0.0012,$ δ=0.03, $\Lambda_{s}=1200$ and for sub-figure (b) are $\beta_{s}=0.76$, $\alpha_{a}=0.264,$ $\alpha_{i}=0.76,$ $\alpha_{u}=0.96,$ $\gamma_{q}=0.0015,$ δ=0.03, $\Lambda_{s}=1200$; other parameter values are listed in Table I.

The predictive competency for the SAIUQR model system (1) requires valid estimation of the system parameters $\gamma_{a}$ (rate of transition from asymptomatic to symptomatic infected individuals), $\gamma_{q}$ (the rate that the quarantine become susceptible), θ (fraction of asymptomatic infectious that become reported symptomatic infectious), and $\xi_{a}$ (rate at which asymptomatic individuals become quarantined). In Fig. 6(a), we plot the reproduction number $R_{0}$ as a function of $\xi_{a}$ and $\gamma_{q}$ for the parameter values in Table I and estimated parameters for the state Jharkhand, to encapsulate the significance of these values in the evolution of COVID-19 outbreak. From Fig. 6(a), it can be observed that the parameters have a little influence on the outbreak of the coronavirus disease as the parameters $\xi_{a}$ and $\gamma_{q}$ have a little contribution for the reproduction number $R_{0}$. In Fig. 6(b), we plot the reproduction number $R_{0}$ as a function of θ and $\gamma_{a}$ for the parameter values in Table I and estimated parameters for the state Jharkhand, to encapsulate the significance of these values in the evolution of COVID-19 outbreak. Figure 6(b) shows that the parameters θ and $\gamma_{a}$ are more influential in increasing the reproduction number $R_{0}$. Thus, to control the outbreak of COVID-19, we must control the parameters θ and $\gamma_{a}$. The correctness of these values relies on the input of medical and biological epidemiologists. Thus, the fraction θ of reported symptomatic infected individuals may be substantially increased by public health reporting measures, with greater efforts to recognize all present cases. Our model simulation reveals the effect of an increase in this fraction θ in the value of the reproduction number $R_{0}$, as evident in Fig. 6(b), for the COVID-19 epidemic in the four states of India.

### C. Short-term prediction

**FIG. 7.**
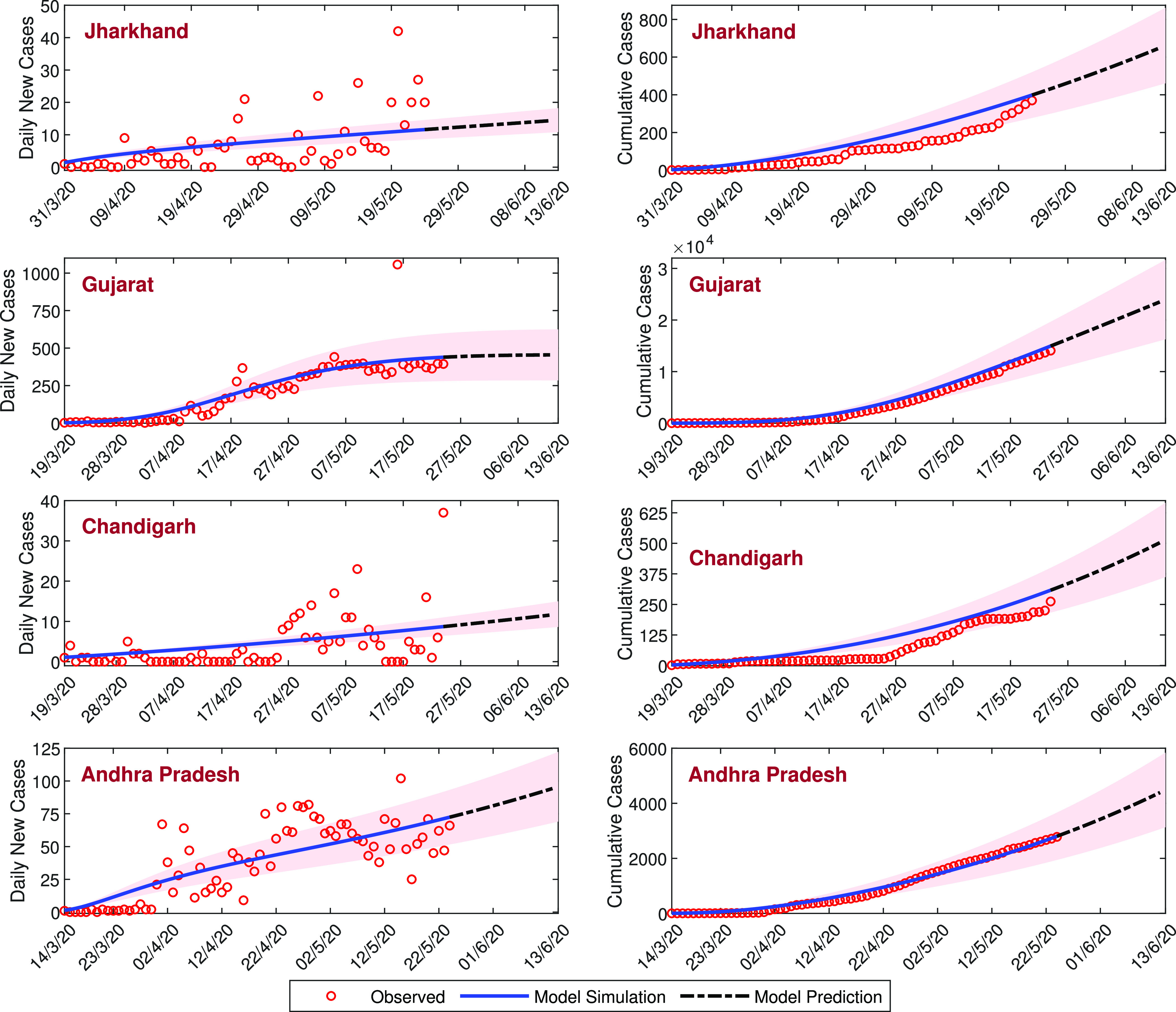
The short-term prediction of the daily new COVID-19 cases (first column) and the cumulative confirmed cases (second column) for the three states of India, namely, Jharkhand, Gujarat, Andhra Pradesh, and one city of India, namely, Chandigarh. The black dotted-dashed curve represents the prediction from May 25, 2020 to June 13, 2020 (20 days). The red shaded region is the standard deviation band of the SAIUQR model simulated curve.

Mathematical modeling of infectious diseases can provide short-term and long-term prediction of the pandemic.^16,18,20,32^ Due to the absence of any licensed vaccines or specific therapeutics, forecasting is of utmost importance for strategies to control and in prevention of the diseases with limited resources. It should be noted here that we can predict the epidemiological traits of SARS-CoV-2 or COVID-19 for short-term only as the Governmental strategies can be altered time to time, resulting in the corresponding changes in the associated parameters of the proposed SAIUQR model. Also, it is true that the scientists are working on drugs and/or effective vaccines against COVID-19 and the presence of such pharmaceutical interventions will substantially change the outcomes.^36^ Thus, in this study, we performed a short-term prediction for our SAIUQR model system (1) using the parameter values in Table I and the estimated parameter values in Table II. Using the observed data up to May 24, 2020, a short-term prediction (for 20 days) has been done for daily new COVID-19 cases (first column) and cumulative confirmed cases (second column), which are presented in Fig. 7. The black dotted-dashed curve represents the short-term prediction of our SAIUQR model from May 25, 2020 to June 13, 2020. The red shaded region is the standard deviation band of our SAIUQR model simulated curve. The standard deviations are computed from the model simulation based on the estimated data. In each of the four states, we plot the standard deviation bands at a standard deviation level above and below the model simulation for different days. The standard deviation band gives an estimation of the deviation of the actual model data. The trend of the predicted daily COVID-19 cases is increasing for all four states of India. Prediction of the SAIUQR model should be regarded as an estimation of the daily infected population and cumulative confirmed cases of the four states of India. From the SAIUQR model simulation, we can predict that the estimated daily newly reported COVID-19 cases on June 13, 2020 will be approximately 15, 454, 12, and 96 in Jharkhand, Gujarat, Chandigarh, and Andhra Pradesh, respectively (see the left column of Fig. 7). Our SAIUQR model simulation predicts that the confirmed cumulative number of cases on June 13, 2020 will be approximately 661, 23 955, 514, and 4487 in Jharkhand, Gujarat, Chandigarh, and Andhra Pradesh, respectively (see the right column of Fig. 7).

## V. DISCUSSION AND CONCLUSION

The SARS-CoV-2 pandemic in India is a potential menace throughout the country due to its exponential growth. Everyday, around 5000–6000 or more new cases are reported from different states and territories of India, which is an alarming situation for the country, which is the second most populated worldwide.^34^ Due to the absence of any licensed vaccine, therapeutics, or treatment and with peculiar epidemiological traits of SARS-CoV-2, one would depend on the qualitative control of the disease rather than complete eradication. During this period of epidemic, when person-to-person transmission is confirmed and the reported cases of SARS-CoV-2 virus are rising throughout the world, prediction is of utmost priority for healthcare planning and to manage the virus with limited resources. Furthermore, mathematical modeling can be a powerful tool in designing strategies to manage the exponentially spreading coronavirus disease in the absence of any antivirals or diagnostic tests.

In this study, we proposed and analyzed a new compartmental mathematical model for SARS-CoV-2 virus to forecast and control the outbreak. In the model formulation, we incorporate the transmission variability of asymptomatic and unreported symptomatic individuals. We also incorporate the symptomatic infected population who are reported by the public health services. We assume that the reported infected individuals will no longer be associated with the infection as they are isolated and moved to the hospital or Intensive Care Unit (ICU). In our model, we incorporate the constant transmission rate in the early exponential growth phase of the SARS-CoV-2 disease as identified in Refs. 18 and 29. We model the role of the government imposed restrictions for the public in India, beginning on March 25, 2020, as a time-dependent decaying transmission rate after March 25, 2020. But, due to less stringent lockdown, the disease transmission rate is exponentially increasing; we were able to fit our model simulations to the Indian reported cases data up to May 24, 2020 with accuracy.

We fit our SAIUQR model for the daily confirmed cases and cumulative confirmed cases of the four different states of India, namely, Jharkhand, Andhra Pradesh, Chandigarh, and Gujarat with data up to May 24, 2020. The estimated model parameters for different states of India are given in Table II and the corresponding initial population size is listed in Table III. It can be observed that the basic reproduction number for four different states of India, namely, Jharkhand, Andhra Pradesh, Chandigarh, and Gujarat are 1.6877, 1.2435, 1.4775, and 1.8830, respectively, which demonstrates that the disease transmission rate is quite high, indicating the substantial outbreak of the COVID-19 disease. This higher value of reproduction number $R_{0}$ captures the outbreak of COVID-19 phenomena in India. Based on the estimated parameter values, our model simulation suggests that the rate of disease transmission needs to be controlled, otherwise India will enter in stage-3 of SARS-CoV-2 disease transmission within a short period of time.

Based on the estimated model parameters, we have validated our detailed analytical findings. Our proposed SAIUQR model has two biologically feasible singular points, namely, infection-free steady state $E^{0}$ and a unique endemic steady state $E^{*}$, and they become locally asymptotically stable for $R_{0}<1$ and $R_{0}>1$, respectively. Analytically, we have shown that the infection-free steady state $E^{0}$ of the SAIUQR model (1) is globally asymptotically stable for $R_{0}<1.$ We also showed that the SAIUQR model (1) experiences transcritical bifurcation at the threshold parameter $R_{0}=1$, which has been shown in Fig. 4. Blue curves and red curves in Fig. 3 represent the local asymptotic stability as well as global asymptotic stability of the infection-free steady state $E^{0}$ for $R_{0}<1$ and endemic steady state $E^{*}$ for $R_{0}>1$, respectively.

The calibrated model is then utilized for short-term predictions in the four different states of India. Our SAIUQR model performs well in the case of all four states of India, namely, Jharkhand, Chandigarh, Gujarat, and Andhra Pradesh for daily confirmed cases and cumulative confirmed cases. However, the increasing (or exponential) pattern of daily new cases and cumulative confirmed cases of SARS-CoV-2 is well captured by our proposed model for all four states of India, which has been shown in Fig. 2. Our model simulation showed a short-term prediction for 20 days (from May 25, 2020 to June 13, 2020) for daily confirmed cases and cumulative confirmed cases of the four states of India. The short-term prediction for the four states of India will demonstrate the increasing pattern of the daily and cumulative cases in the near future (see Fig. 7). From the simulation, our model predicts that on June 13, 2020, the daily confirmed cases of COVID-19 of the four states of India, namely, Jharkhand, Gujarat, Chandigarh, and Andhra Pradesh will be 15, 454, 12, and 96, respectively (see the left column of Fig. 7). Similarly, from the simulation, our model predicts that on June 13, 2020, the cumulative confirmed cases of COVID-19 of the four states of India, namely, Jharkhand, Gujarat, Chandigarh, and Andhra Pradesh will be 661, 23 955, 514, and 4487, respectively (see the right column of Fig. 7).

It is worth mentioning that the scientists or clinicians are working for an effective vaccine or therapeutics to eradicate and/or control the outbreak of the SARS-CoV-2 disease, and the existence of such pharmaceutical interventions will substantially change the outcomes.^36,37^ Thus, in this study, we are mainly focusing on short-term predictions for the COVID-19 pandemic and subsequently, there would be a very little chance to alter in the corresponding parametric space. The framework of our present compartmental model provides some significant insights into the dynamics and forecasting of the spread and control of COVID-19. Moreover, our model simulation suggests that quarantine, reported, and unreported symptomatic individuals as well as government intervention polices like media effect, lockdown, and social distancing can play a key role in mitigating the transmission of COVID-19.

## Data Availability

All data used in this work have been obtained from official sources.^34^ The data that support the findings of this study are available from the corresponding author upon reasonable request.

## APPENDIX A: PROOF OF THEOREM 3.1

Proof. To prove the positivity of system (1), we show that any solution initiating from the non-negative octant $R_{+}^{6}$ remains positive for all t>0. In order to do this, we have to prove that on each hyperplane bounding the non-negative octant, the vector field points into $R_{+}^{6}$. For our system (1), we observe that

$$\begin{array}{rl} \frac{dS}{dt}{|}_{S=0} & =\Lambda_{s}+\rho_{s}\gamma_{q}Q\;\geq \;0,\\ \frac{dA}{dt}{|}_{A=0} & =\beta_{s}S(\alpha_{i}\frac{I}{N}+\alpha_{u}\frac{U}{N})\;\geq \;0,\\ \frac{dI}{dt}{|}_{I=0} & =\theta \gamma_{a}A+(1-\rho_{s})\gamma_{q}Q\;\geq \;0,\\ \frac{dU}{dt}{|}_{U=0} & =(1-\theta )\gamma_{a}A\;\geq \;0,\\ \frac{dQ}{dt}{|}_{Q=0} & =\xi_{a}A\;\geq \;0,\\ \frac{dR}{dt}{|}_{R=0} & =\eta_{u}U+\eta_{i}I+\eta_{a}A\;\geq \;0. \end{array}$$

Therefore, the positivity of the solutions starting in the interior of $R_{+}^{6}$ is assured. $R_{+}^{6}$ is positively invariant set of the SAIUQR model system (1).

## APPENDIX B: PROOF OF THEOREM 3.2

Proof. To prove the boundedness of the SAIUQR system (1), we add all model equations, which gives N=S+A+I+U+Q+R. Taking the differentiation gives

$$\frac{dN}{dt}=\Lambda_{s}-\delta N,$$

which gives

$$\underset{t\to \infty }{lim sup}N(t)\leq \frac{\Lambda_{s}}{\delta }.$$

Without any loss of generality, we can assume that $\underset{t\to \infty }{lim sup}S(t)\leq \frac{\Lambda_{s}}{\delta },$ $\underset{t\to \infty }{lim sup}A(t)\leq \frac{\Lambda_{s}}{\delta },$ $\underset{t\to \infty }{lim sup}I(t)\leq \frac{\Lambda_{s}}{\delta },$ $\underset{t\to \infty }{lim sup}U(t)\leq \frac{\Lambda_{s}}{\delta },$ $\underset{t\to \infty }{lim sup}Q(t)\leq \frac{\Lambda_{s}}{\delta }$, and $\underset{t\to \infty }{lim sup}R(t)\leq \frac{\Lambda_{s}}{\delta }.$ Thus, we have a bounded set

$$\Omega =\{(S,A,I,U,Q,R)\in R_{+}^{6}:0\leq S,A,I,U,Q,R\leq \frac{\Lambda_{s}}{\delta }\},$$

which is also a positively invariant set with respect to the SAIUQR model (1). Thus, any solution trajectory starting from an interior point of $R_{+}^{6}$ ultimately enters into the region Ω and remains there for all finite time. This result indicates that none of the individuals grow unboundedly or exponentially for a finite time window.

## APPENDIX C: PROOF OF THEOREM 3.3

Proof. The variational matrix around the infection-free steady state $E^{0}$ for the SAIUQR model system (1) is given by

$$J_{E^{0}}=\left(\begin{array}{cccccc} -\delta & -\beta_{s}\alpha_{a} & -\beta_{s}\alpha_{i} & -\beta_{s}\alpha_{u} & \rho_{s}\gamma_{q} & 0\\ 0 & \beta_{s}\alpha_{a}-(\xi_{a}+\gamma_{a}+\eta_{a}+\delta ) & \beta_{s}\alpha_{i} & \beta_{s}\alpha_{u} & 0 & 0\\ 0 & \theta \gamma_{a} & -(\eta_{i}+\delta ) & 0 & (1-\rho_{s})\gamma_{q} & 0\\ 0 & (1-\theta )\gamma_{a} & 0 & -(\eta_{u}+\delta ) & 0 & 0\\ 0 & \xi_{a} & 0 & 0 & -(\gamma_{q}+\delta ) & 0\\ 0 & \eta_{a} & \eta_{i} & \eta_{u} & 0 & -\delta \end{array}\right).$$

The above Jacobian matrix $J_{E^{0}}$ has two repeated eigenvalues which are −δ, while the other four eigenvalues are the roots of the following characteristics equation $|J_{E^{0}}-\lambda I|=0$:

$$\begin{array}{rl} & (\xi_{a}+\gamma_{a}+\eta_{a}+\delta +\lambda )(\eta_{i}+\delta +\lambda )(\eta_{u}+\delta +\lambda )(\gamma_{q}+\delta +\lambda )\\ & \;-\beta_{s}\alpha_{a}(\eta_{i}+\delta +\lambda )(\eta_{u}+\delta +\lambda )(\gamma_{q}+\delta +\lambda )\\ & \;-\theta \gamma_{a}\beta_{s}\alpha_{i}(\eta_{u}+\delta +\lambda )(\gamma_{q}+\delta +\lambda )\\ & \;-(1-\theta )\gamma_{a}\beta_{s}\alpha_{u}(\eta_{i}+\delta +\lambda )(\gamma_{q}+\delta +\lambda )\\ & \;-(1-\rho_{s})\gamma_{q}\xi_{a}\beta_{s}\alpha_{i}(\eta_{u}+\delta +\lambda )\;=\;0, \end{array}$$

which can be rewritten in the following form:

$$\begin{array}{rl} & \frac{\beta_{s}\alpha_{a}}{\xi_{a}+\gamma_{a}+\eta_{a}+\delta +\lambda }+\frac{\theta \gamma_{a}\beta_{s}\alpha_{i}}{\left(\xi_{a}+\gamma_{a}+\eta_{a}+\delta +\lambda )(\eta_{i}+\delta +\lambda \right)}\\ & \;+\frac{(1-\theta )\gamma_{a}\beta_{s}\alpha_{u}}{\left(\xi_{a}+\gamma_{a}+\eta_{a}+\delta +\lambda )(\eta_{u}+\delta +\lambda \right)}\\ & \;+\frac{(1-\rho_{s})\gamma_{q}\xi_{a}\beta_{s}\alpha_{i}}{\left(\xi_{a}+\gamma_{a}+\eta_{a}+\delta +\lambda )(\eta_{i}+\delta +\lambda )(\gamma_{q}+\delta +\lambda \right)}\;=\;1. \end{array}$$

Denote the above expression as the following:

$$\begin{array}{rl} m_{1}(\lambda ) & =\frac{\beta_{s}\alpha_{a}}{\xi_{a}+\gamma_{a}+\eta_{a}+\delta +\lambda }\\ & \;+\frac{\theta \gamma_{a}\beta_{s}\alpha_{i}}{\left(\xi_{a}+\gamma_{a}+\eta_{a}+\delta +\lambda )(\eta_{i}+\delta +\lambda \right)}\\ & \;+\frac{(1-\theta )\gamma_{a}\beta_{s}\alpha_{u}}{\left(\xi_{a}+\gamma_{a}+\eta_{a}+\delta +\lambda )(\eta_{u}+\delta +\lambda \right)}\\ & \;+\frac{(1-\rho_{s})\gamma_{q}\xi_{a}\beta_{s}\alpha_{i}}{\left(\xi_{a}+\gamma_{a}+\eta_{a}+\delta +\lambda )(\eta_{i}+\delta +\lambda )(\gamma_{q}+\delta +\lambda \right)},\\ & =m_{11}(\lambda )+m_{22}(\lambda )+m_{33}(\lambda )+m_{44}(\lambda )\;\;\;\text{(say)}. \end{array}$$

Substitute λ=x+iy, and we know that Re(λ)≥0; then, the above expression leads to

$$\begin{array}{rl} |m_{11}(\lambda )| & \leq \frac{\beta_{s}\alpha_{a}}{|\xi_{a}+\gamma_{a}+\eta_{a}+\delta +\lambda |}\;\leq \;m_{11}(x)\\ \; & \leq \;m_{11}(0),\\ |m_{22}(\lambda )| & \leq \frac{\theta \gamma_{a}\beta_{s}\alpha_{i}}{|\xi_{a}+\gamma_{a}+\eta_{a}+\delta +\lambda ||\eta_{i}+\delta +\lambda |}\;\leq \;m_{22}(x)\\ \; & \leq \;m_{22}(0),\\ |m_{33}(\lambda )| & \leq \frac{(1-\theta )\gamma_{a}\beta_{s}\alpha_{u}}{|\xi_{a}+\gamma_{a}+\eta_{a}+\delta +\lambda ||\eta_{u}+\delta +\lambda |}\\ \; & \leq \;m_{33}(x)\;\leq \;m_{33}(0),\\ |m_{44}(\lambda )| & \leq \frac{(1-\rho_{s})\gamma_{q}\xi_{a}\beta_{s}\alpha_{i}}{|\xi_{a}+\gamma_{a}+\eta_{a}+\delta +\lambda ||\eta_{i}+\delta +\lambda ||\gamma_{q}+\delta +\lambda |}\\ \; & \leq \;m_{44}(x)\;\leq \;m_{44}(0). \end{array}$$

Thus, $m_{11}(0)+m_{22}(0)+m_{33}(0)+m_{44}(0)\;=\;m_{1}(0)\;=\;R_{0}<1,$ which gives $m_{1}(\lambda )\leq 1.$ Therefore, for $R_{0}<1$, all eigenvalues of the characteristic equation $m_{1}(\lambda )=1$ are real or have negative real parts. Thus, for $R_{0}<1$, all eigenvalues are negative, and hence, the infection-free steady state $E^{0}$ is locally asymptotically stable. Now, if we consider $R_{0}>1$, that is, $m_{1}(0)>1$, then

$$\lim_{\lambda \to \infty }m_{1}(\lambda )=0;$$

there exists $\lambda_{1}^{*}>0$ in such a way that $m_{1}(\lambda_{1}^{*})=1.$ This indicates that there exists non-negative eigenvalue $\lambda_{1}^{*}>0$ for the variational matrix $J_{E^{0}}$. Hence, the infection-free steady state $E^{0}$ is unstable for $R_{0}>1$.

## APPENDIX D: PROOF OF THEOREM 3.4

Proof. To prove the global asymptotic stability of the infection-free steady state $E^{0}(S^{0},A^{0},Q^{0},I^{0},U^{0},R^{0})$, we can rewrite the SAIUQR model (1) in the following form:

$$\begin{array}{rl} \frac{dX}{dt} & =F(X,V),\\ \frac{dV}{dt} & =G(X,V),\;G(X,0)=0, \end{array}$$

where $X=(S,R)\in R^{2}$ represents (its components) the number of susceptible or uninfected individuals and $V=(A,I,U,Q)\in R^{4}$ represents (its components) the number of infected individuals incorporating asymptomatic, quarantine, infectious, etc. $E^{0}=(X^{*},0)$ designates the infection-free steady state for the SAIUQR model system (1). For the compartmental model (1), F(X,V) and G(X,V) are defined as follows:

$$F(X,V)=\left(\begin{array}{c} \Lambda_{s}-\beta_{s}\frac{S}{N}(\alpha_{a}A+\alpha_{i}I+\alpha_{u}U)+\rho_{s}\gamma_{q}Q-\delta S\\ \eta_{u}U+\eta_{i}I+\eta_{a}A-\delta R \end{array}\right)$$

and

$$G(X,V)=\left(\begin{array}{c} \beta_{s}\frac{S}{N}(\alpha_{a}A+\alpha_{i}I+\alpha_{u}U)-(\xi_{a}+\gamma_{a})A-\eta_{a}A-\delta A\\ \theta \gamma_{a}A+(1-\rho_{s})\gamma_{q}Q-\eta_{i}I-\delta I\\ (1-\theta )\gamma_{a}A-\eta_{u}U-\delta U\\ \xi_{a}A-\gamma_{q}Q-\delta Q \end{array}\right).$$

From the above expression of G(X,V), it is clear that G(X,0)=0. The following two conditions (C1) and (C2) must be met to assure the global asymptotic stability: (C1) For $\frac{dX}{dt}=F(X,0),$ $X^{*}$ is globally asymptotically stable, (C2) $G(X,V)=BV-\hat{G}(X,V),$ $\hat{G}(X,V)\geq 0$ for (X,V)∈Ω, where $B=D_{I}G(X^{*},0)$ is an M-matrix (the non-diagonal components are non-negative) and in the region Ω, the SAIUQR model system (1) is biologically feasible. The compartmental model (1) stated in the condition (C1) can be expressed as

$$\frac{d}{dt}\left(\begin{array}{c} S\\ R \end{array}\right)\;=\;\left(\begin{array}{c} \Lambda_{s}-\delta S\\ -\delta R \end{array}\right).$$

Analytically solving the above system of equations, we obtain that $S(t)=\frac{\Lambda_{s}}{\delta }+\exp(-\delta t)(S(0)-\frac{\Lambda_{s}}{\delta })$ and R(t)=exp⁡(−δt)R(0). Considering t→∞, $S(t)=\frac{\Lambda_{s}}{\delta }$ and R(t)→0. Thus, $X^{*}$ is globally asymptotically stable for $\frac{dX}{dt}=F(X,0).$ Thus, the first condition (C1) holds for system (1). Now the matrices B and $\hat{G}(X,V)$ for the SAIUQR model system (1) can be expressed as

$$\begin{array}{rl} B & =\left(\begin{array}{cccc} -(\xi_{a}+\gamma_{a}+\eta_{a}+\delta )+\alpha_{a}\beta_{s} & \alpha_{i}\beta_{s} & \alpha_{u}\beta_{s} & 0\\ \theta \gamma_{a} & -(\eta_{i}+\delta ) & 0 & (1-\rho_{s})\gamma_{q}\\ (1-\theta )\gamma_{a} & 0 & -(\eta_{u}+\delta ) & 0\\ \xi_{a} & 0 & 0 & -(\gamma_{q}+\delta ) \end{array}\right),\\ \hat{G}(X,V) & =\left(\begin{array}{c} \alpha_{a}\beta_{s}A(1-\frac{S}{N})+\alpha_{i}\beta_{s}I(1-\frac{S}{N})+\alpha_{u}\beta_{s}U(1-\frac{S}{N})\\ 0\\ 0\\ 0 \end{array}\right). \end{array}$$

It is clear that B is a M-matrix as all its non-diagonal components are non-negative. Also, $\hat{G}(X,V)\geq 0$ in the region Ω as S(t)≤N(t). Also, we showed that $X^{*}$ is a globally asymptotically stable steady state of the system $\frac{dX}{dt}=F(X,0)$. Therefore, the infection-free steady state $E^{0}$ of the SAIURQ model (1) is globally asymptotically stable in the region Ω for $R_{0}<1.$

## APPENDIX E: PROOF OF THEOREM 3.5

Proof. Now, we use the theory of center manifold to investigate the local asymptotic stability of the interior equilibrium point $E^{*}(S^{*},A^{*},Q^{*},I^{*},U^{*},R^{*})$ by considering the disease transmission rate $\beta_{s}$ as a bifurcation parameter, $\beta_{s}=\beta_{s}^{c}$ corresponding to $R_{0}=1,$ is

$$\begin{array}{r} \beta_{s}^{c}=\frac{\left(\eta_{u}+\delta )(\gamma_{q}+\delta )(\eta_{i}+\delta )(\xi_{a}+\gamma_{a}+\eta_{a}+\delta \right)}{\alpha_{a}(\eta_{u}+\delta )(\gamma_{q}+\delta )(\eta_{i}+\delta )+(1-\theta )\alpha_{u}\gamma_{a}(\gamma_{q}+\delta )(\eta_{i}+\delta )+\alpha_{i}\left\{\theta \gamma_{a}(\gamma_{q}+\delta )+(1-\rho_{s})\xi_{a}\gamma_{a}\right\}(\eta_{u}+\delta )}. \end{array}$$

The variational matrix of the SAIUQR model (1) at $\beta_{s}=\beta_{s}^{c}$, denoted by $J_{E^{0}}$ has the right eigenvector associated, with zero being the eigenvalue given by $\omega ={\left[\omega_{1},\;\omega_{2},\;\omega_{3},\;\omega_{4},\;\omega_{5},\;\omega_{6}\right]}^{T}$, where

$$\begin{array}{rl} \omega_{1} & =\frac{\omega_{2}}{\delta }\left[-\frac{(1-\theta )\gamma_{a}\beta_{s}\alpha_{u}}{\eta_{u}+\delta }+\frac{\rho_{s}\gamma_{q}\xi_{a}}{\eta_{q}+\delta }-(\xi_{a}+\gamma_{a}+\eta_{a}+\delta \right)\\ & \;+\frac{\beta_{s}\alpha_{u}(1-\theta )\gamma_{a}}{\eta_{u}+\delta }],\\ \omega_{2} & =\omega_{2}>0,\;\omega_{4}=\frac{(1-\theta )\gamma_{a}\omega_{2}}{\eta_{u}+\delta },\;\omega_{5}\;=\;\frac{\xi_{a}\omega_{2}}{\eta_{q}+\delta },\\ \omega_{3} & =\frac{\omega_{2}}{\beta_{s}\alpha_{i}}\left[(\xi_{a}+\gamma_{a}+\eta_{a}+\delta )-\beta_{s}\alpha_{a}-\frac{\beta_{s}\alpha_{u}(1-\theta )\gamma_{a}}{\eta_{u}+\delta }\right],\\ \omega_{6} & =\frac{\omega_{2}}{\delta }[\eta_{a}+\frac{\eta_{u}(1-\theta )\gamma_{a}}{\eta_{u}+\delta }+\frac{\eta_{i}((\xi_{a}+\gamma_{a}+\eta_{a}+\delta )-\beta_{s}\alpha_{a})}{\beta_{s}\alpha_{i}}\\ & \;-\frac{\eta_{i}\beta_{s}\alpha_{u}(1-\theta )\gamma_{a}}{\beta_{s}\alpha_{i}(\eta_{u}+\delta )}]. \end{array}$$

Similarly, at the threshold $\beta_{s}=\beta_{s}^{c}$, the variational matrix $J_{E^{0}}$ has the left eigenvector associated, with zero being the eigenvalue given by $\upsilon =[\upsilon_{1},\;\upsilon_{2},\;\upsilon_{3},\;\upsilon_{4},\;\upsilon_{5},\;\upsilon_{6}]$, where

$$\begin{array}{rl} \upsilon_{1} & =0,\;\upsilon_{6}=0,\;\upsilon_{2}=\upsilon_{2}>0,\\ \upsilon_{3} & =\frac{\beta_{s}\alpha_{i}\upsilon_{2}}{\eta_{i}+\delta },\;\upsilon_{4}=\frac{\beta_{s}\alpha_{u}\upsilon_{2}}{\eta_{u}+\delta },\\ \upsilon_{5} & =\frac{\upsilon_{2}}{\xi_{a}}[(\xi_{a}+\gamma_{a}+\eta_{a}+\delta )-\beta_{s}\alpha_{a}-\frac{\theta \gamma_{a}\beta_{s}\alpha_{i}}{\eta_{i}+\delta }\\ & \;-\frac{\beta_{s}\alpha_{u}(1-\theta )\gamma_{a}}{\eta_{u}+\delta }]. \end{array}$$

Let us introduce the notations for the SAIUQR model system (1): $S=x_{1}$, $A=x_{2}$, $I=x_{3}$, $U=x_{4}$, $Q=x_{5}$, $R=x_{6}$, and $\frac{dx_{i}}{dt}=f_{i}$, where i=1,2,…,6. Now, we compute the following nonzero second order partial derivatives of $f_{i}$ at the infection-free steady state $E^{0}$ and obtain

$$\begin{array}{rl} \frac{\partial^{2}f_{2}}{\partial x_{2}\partial x_{3}} & =-\beta_{s}(\alpha_{a}+\alpha_{i})\frac{\Lambda_{s}}{\delta },\;\frac{\partial^{2}f_{2}}{\partial x_{2}\partial x_{4}}=-\beta_{s}(\alpha_{a}+\alpha_{u})\frac{\Lambda_{s}}{\delta },\\ \frac{\partial^{2}f_{2}}{\partial x_{2}\partial x_{5}} & =-\beta_{s}\alpha_{a}\frac{\Lambda_{s}}{\delta },\;\;\;\frac{\partial^{2}f_{2}}{\partial x_{2}\partial x_{6}}\;=\;-\beta_{s}\alpha_{a}\frac{\Lambda_{s}}{\delta },\\ \frac{\partial^{2}f_{2}}{\partial x_{3}\partial x_{4}} & =-\beta_{s}(\alpha_{i}+\alpha_{u})\frac{\Lambda_{s}}{\delta },\;\;\;\frac{\partial^{2}f_{2}}{\partial x_{3}\partial x_{5}}\;=\;-\beta_{s}\alpha_{i}\frac{\Lambda_{s}}{\delta },\\ \frac{\partial^{2}f_{2}}{\partial x_{3}\partial x_{6}} & =-\beta_{s}\alpha_{i}\frac{\Lambda_{s}}{\delta },\;\;\;\frac{\partial^{2}f_{2}}{\partial x_{4}\partial x_{6}}\;=\;-\beta_{s}\alpha_{u}\frac{\Lambda_{s}}{\delta },\\ \frac{\partial^{2}f_{2}}{\partial x_{4}\partial x_{5}} & =-\beta_{s}\alpha_{u}\frac{\Lambda_{s}}{\delta },\;\;\frac{\partial^{2}f_{2}}{\partial x_{2}\partial x_{2}}\;=\;-2\beta_{s}\alpha_{a}\frac{\Lambda_{s}}{\delta },\\ \frac{\partial^{2}f_{2}}{\partial x_{3}\partial x_{3}} & =-2\beta_{s}\alpha_{i}\frac{\Lambda_{s}}{\delta },\;\;\;\frac{\partial^{2}f_{2}}{\partial x_{4}\partial x_{4}}\;=\;-2\beta_{s}\alpha_{u}\frac{\Lambda_{s}}{\delta }. \end{array}$$

The rest of the partial derivatives at the infection-free steady state $E^{0}$ remains zero. Now, we compute the coefficients a and b due to the well-known Theorem 4.1 by Castillo-Chavez and Song^38^ as follows:

$$a=\sum_{i,j,k=1}^{6}\upsilon_{k}\omega_{i}\omega_{j}\frac{\partial^{2}f_{k}(0,0)}{\partial x_{i}\partial x_{j}}$$

and

$$b=\sum_{i,k=1}^{6}\upsilon_{k}\omega_{i}\frac{\partial^{2}f_{k}(0,0)}{\partial x_{i}\partial \beta_{s}}.$$

By substituting the values of all nonzero second order partial derivatives and the left and right eigenvectors from the above analysis at threshold $\beta_{s}=\beta_{s}^{c}$, we have

$$\begin{array}{rl} a & =-\frac{\beta_{s}\upsilon_{2}\Lambda_{s}}{\delta }(\omega_{2}\omega_{3}(\alpha_{a}+\alpha_{i})+\omega_{2}\omega_{4}(\alpha_{a}+\alpha_{u})\\ & \;+\omega_{2}\omega_{5}\alpha_{a}+\omega_{2}\omega_{6}\alpha_{a}+\omega_{3}\omega_{4}(\alpha_{i}+\alpha_{u})+\omega_{3}\omega_{5}\alpha_{i}\\ & \;+\omega_{3}\omega_{6}\alpha_{i}+\omega_{4}\omega_{6}\alpha_{u}+\omega_{4}\omega_{5}\alpha_{u}+2\omega_{2}^{2}\alpha_{a}\\ & \;+2\omega_{3}^{2}\alpha_{i}+2\omega_{4}^{2}\alpha_{u}) \end{array}$$

and

$$b=\frac{\upsilon_{2}\Lambda_{s}}{\delta }(\omega_{2}\alpha_{a}+\omega_{3}\alpha_{i}+\omega_{4}\alpha_{u}).$$

From the above expressions, it can be observed that a<0 and b>0; therefore, by Remark 1 of the well-known Theorem 4.1 by Castillo-Chavez and Song,^38^ and by Khajanchi *et al.*,^39^ a transcritical bifurcation occurs at the basic reproduction number $R_{0}=1$, and the interior equilibrium point $E^{*}$ is locally asymptotically stable for $R_{0}>1$.
